# Transcriptome profiling of *Malus sieversii* under freezing stress after being cold-acclimated

**DOI:** 10.1186/s12864-021-07998-0

**Published:** 2021-09-21

**Authors:** Ping Zhou, Xiaoshuang Li, Xiaojie Liu, Xuejing Wen, Yan Zhang, Daoyuan Zhang

**Affiliations:** 1 State Key Laboratory of Desert and Oasis Ecology, Xinjiang Institute of Ecology and Geography, Chinese Academy of Sciences, Urumqi, 830011 China; 2 University of Chinese Academy of Sciences, Beijing, 100049 China; 3 Turpan Eremophytes Botanical Garden, Chinese Academy of Sciences, Turpan, 838008 China

**Keywords:** *Malus sieversii*, Freezing stress, Photosynthesis, Plant hormone signal transduction, Sugar and starch metabolism, Transcription factor

## Abstract

**Background:**

Freezing temperatures are an abiotic stress that has a serious impact on plant growth and development in temperate regions and even threatens plant survival. The wild apple tree (*Malus sieversii*) needs to undergo a cold acclimation process to enhance its freezing tolerance in winter. Changes that occur at the molecular level in response to low temperatures are poorly understood in wild apple trees.

**Results:**

Phytohormone and physiology profiles and transcriptome analysis were used to elaborate on the dynamic response mechanism. We determined that JA, IAA, and ABA accumulated in the cold acclimation stage and decreased during freezing stress in response to freezing stress. To elucidate the molecular mechanisms of freezing stress after cold acclimation, we employed single molecular real-time (SMRT) and RNA-seq technologies to study genome-wide expression profiles in wild apple. Using the PacBio and Illumina platform, we obtained 20.79G subreads. These reads were assembled into 61,908 transcripts, and 24,716 differentially expressed transcripts were obtained. Among them, 4410 transcripts were differentially expressed during the whole process of freezing stress, and these were examined for enrichment via GO and KEGG analyses. Pathway analysis indicated that “plant hormone signal transduction”, “starch and sucrose metabolism”, “peroxisome” and “photosynthesis” might play a vital role in wild apple responses to freezing stress. Furthermore, the transcription factors *DREB1/CBF, MYC2, WRKY70, WRKY71, MYB4* and *MYB88* were strongly induced during the whole stress period.

**Conclusions:**

Our study presents a global survey of the transcriptome profiles of wild apple trees in dynamic response to freezing stress after two days cold acclimation and provides insights into the molecular mechanisms of freezing adaptation of wild apple plants for the first time. The study also provides valuable information for further research on the antifreezing reaction mechanism and genetic improvement of *M. sieversii* after cold acclimation.

**Supplementary Information:**

The online version contains supplementary material available at 10.1186/s12864-021-07998-0.

## Background

Central Asian mountains are particularly important and have been listed as a global biodiversity hotspot. These hotspots contain ancestors of some domestic fruit, including apples. *Malus sieversii* (also called “Xinjiang wild apple”), the ancestor of domesticated apple [1], is mainly distributed in the Tianshan Mountains of China, Kazakhstan, Kyrgyzstan, and Uzbekistan. *M. sieversii* is a germplasm resource with excellent cold resistance traits and is usually used as a popular rootstock for breeding cold-resistant domesticated apple in China [2, 3]. Therefore, it is of great significance to understand the cold resistance mechanism of wild apple for its further protection and utilization.

As an abiotic stress, low temperature not only affects the geographical distribution of crops, but also has a negative impact on crop yield and quality. Cold stresses are divided into chilling (0–15 °C) and freezing (< 0 °C) damage, can lead to tissue damage and even plant death, and often bring large economic losses to agricultural production [4]. When plants are exposed to low temperatures, the cell membrane is the main site of cold-induced damage, which triggers a series of physiological and biochemical reactions [5]. After exposure to low temperature for a period of time, many plants can improve frost resistance, a process called cold acclimation (CA) [6]. During cold acclimation, plants are able to sense environmental temperature changes, and then trigger a series of protective mechanisms, including changing the composition, structure and function of membrane; synthesis of cryoprotectant molecules, such as soluble sugar, protein and proline; increase the activity of protective enzymes [7]. CA can also induce the expression of some cold-induced genes during the stress period, and these genes likely play a role in stabilizing the cell membrane against freezing injury [8]. For example, CBF/DREB1 protein from Arabidopsis transcription factor family has been identified to control the expression of a regulon of cold-induced genes that increase plant freezing tolerance [9]. In apple (*Malus domestica*), homologue MdCIbHLH1 of Arabidopsis ICE1 contributes to improved cold tolerance by directly activating CBF genes [10]. Furthermore, it is known that the MYB-bHLH-WDR complex, especially the bHLH and MYB components, is involved in modulating anthocyanin accumulation at low temperatures [11]. Additionally, the accumulation of sucrose and other monosaccharides during cold acclimation also contributes to membrane stability because these molecules can protect the membrane from freezing damage in vitro [12]. The entire CA process in nature typically requires much time and is easily affected by climate fluctuations. However, with global warming, the winters and early springs in temperate regions are becoming progressively milder, and temperature patterns are becoming increasingly irregular. This increases the frequency of temperature changes that may cause premature subzero temperatures, thereby subsequent increasing the risk of freezing injury [13]. Additionally, changing phenological patterns, such as an early start of growing season and early flowering time [14], consistent with climate warming, it may increase the risk of tissue damage caused by frost. The likelihood of such scenarios is typically high during early spring. Therefore, it is very important to understand the mechanism of freezing stress after cold acclimation to protect and utilize plants. However, little is known about the molecular mechanism of freezing stress after a short period of cold acclimation in *M. sieversii.*

According to previous research, such as on *Hordeum vulgare* [15], *Arabidopsis thaliana* [16], *Glycine max* [17], and *Camellia sinensis* [18], the molecular mechanism of plants responding to freezing stress has been explained. Transcriptome sequencing was used to obtain differentially expressed genes (DEGs) and further explore the role of related signaling pathways and candidate genes to explain the changes in the plant response to freezing stress. For instance, strawberry (*Fragaria×ananassa*) responds to freezing stress through several pathways: flavonoid biosynthesis, plant hormone signal transduction, MAPK (mitogen-activated protein kinase) signaling, starch and sucrose metabolism, circadian rhythm [19]. A previous transcriptome study identified 4173 and 7734 DEGs in two apple cultivars, respectively, during the chilling and freezing treatments [20]. Oil rapeseed (*Brassica napus*) responds to low-temperature stress through two conserved (primary metabolism and plant hormone signal transduction pathway) and two novel (plant-pathogen interaction and circadian rhythm pathway) signaling pathways [21]. Transcriptomic analysis of *Magnolia wufengensis* under cold stress showed that the response mechanism was related to photosynthesis, and plant hormone signal transduction, primary and secondary metabolism pathways [22].

RNA-seq based on the Illumina platform is a powerful method for investigating gene expression profiles and revealing signal transduction pathways in a wide range of biological systems [23]. In recent years, RNA-seq technology has been widely used to clarify the response of various plants to low temperature stress, including *C. sinensis* [24], and *A. thaliana* [25]. In apple (*Malus domestica*), comparative transcriptome analysis revealed 377 commonly upregulated and 211 commonly downregulated DEGs involved in the crosstalk between drought, cold and high-salinity stress [26]. Single molecule real-time (SMRT) sequencing based on PacBio (Pacific Biosciences) platform can capture the full length of transcripts, which provides a simpler and more accurate method for gene annotation and isoform identification. The third generation sequencing technology represented by PacBio has the advantage of long read length [27]. PacBio and RNA-seq are highly complementary to each other. To obtain an overall view of the molecular regulation during freezing stress after the CA process, we combined PacBio sequencing and RNA-seq to investigate the transcriptome of *M. sieversii* seedlings at low temperatures. This study will provide a theoretical basis for freezing stress resistance and lay a foundation for understanding the cold response molecular mechanism in *M. sieversii*, which provides a rich genetic resource for further research investigating the freezing response in apple and other woody plants.

## Results

### Physiological and plant hormone changes of *M. sieversii* under low temperature stress

**Fig. 1:**
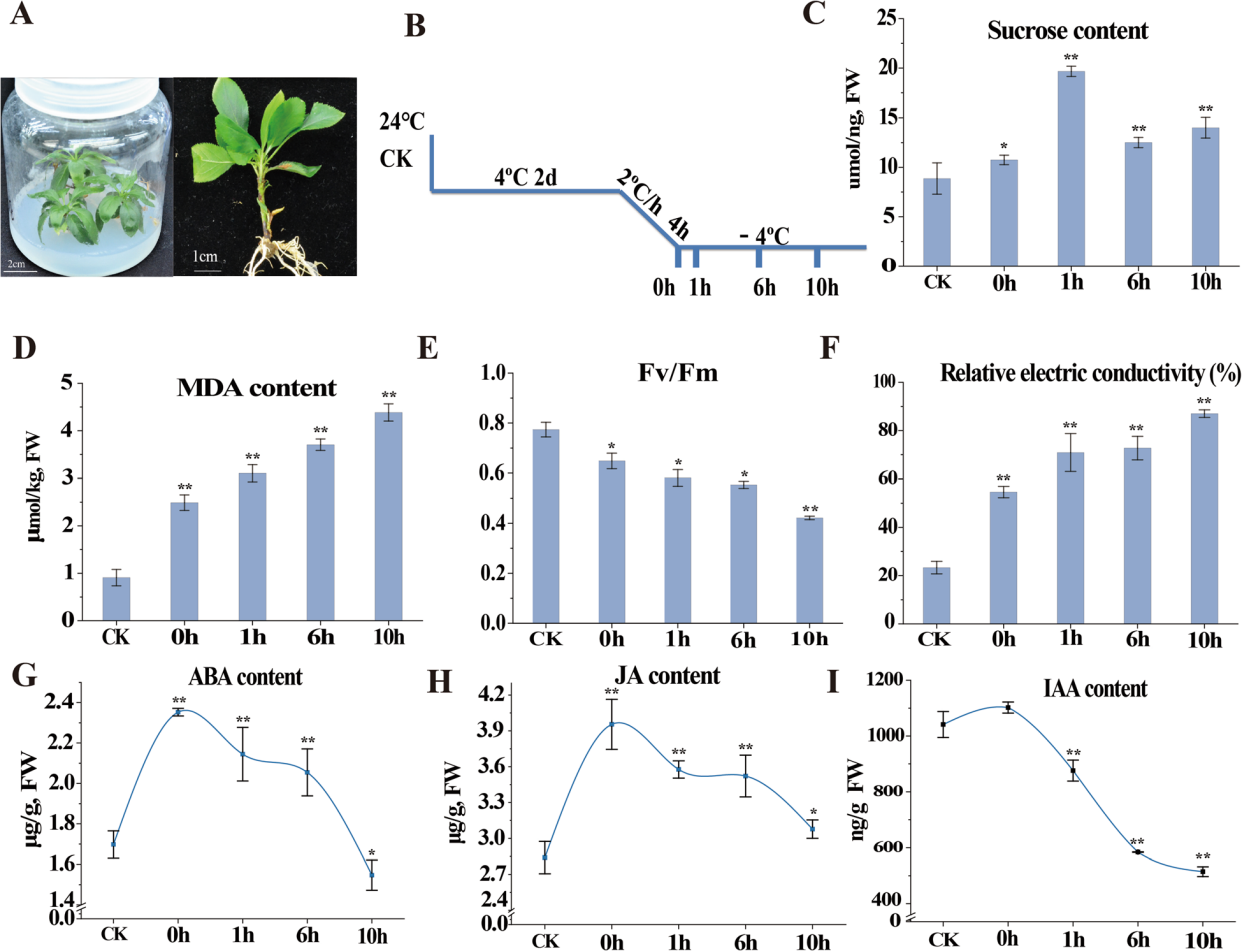
Processing methods and physiological and biochemical indicators after cold treatments at various time points. (A) Two-month-old wild apple seedlings growing in tissue culture bottles (left); harvested leaf (right); (B) Treatment process and sampling time points; (C) sucrose content; (D) MDA content; (E) Fv/Fm values; (F) REC values; (G) endogenous ABA content; (H) endogenous JA content; and (I) endogenous IAA content. Bars represent the mean ± SD (*n* = 3). Accumulation levels were analyzed using a T-test. Within each figure, asterisks above the bars indicate statistical significance (*p < 0.05; **p < 0.01)

In this study, 8-week-old apple plantlets were used as experimental materials to study the transcriptomic and physiological changes of *M. sieversii* under freezing stress (Fig. 1A). The well-developed wild apple seedlings were cold acclimated at 4 °C in a chamber for two days. Subsequently, the temperature was gradually lowered from 4 °C to − 4 °C (2 °C/h) for 4 h, and samples were collected at 0, 1, 6, and 10 h intervals at − 4 °C (Fig. 1B). To evaluate the physiological damage caused by low temperature, we measured relative electrolyte leakage (REC) values, maximum photosynthetic efficiency (Fv/Fm) values, malondialdehyde (MDA) content, and sucrose content. The plants treated at − 4 °C showed a relatively higher sucrose content, which was approximately 200% higher during the 1 h interval (Fig. 1C). Furthermore, under low-temperature treatment, the Fv/Fm values were significantly reduced compared with those of the control (Fig. 1E). In addition, the REC values and MDA production in the treated group were relatively higher than those in the control (Fig. 1D, F), indicating that plasma membranes may be damaged under freezing stress. Low-temperature treatment significantly affected the endogenous hormone content of Xinjiang wild apple leaves. Compared with the control, the contents of endogenous jasmonic acid (JA) and abscisic acid (ABA) after cold acclimation (0 h) increased significantly. Compared with cold acclimation (0 h), the contents of endogenous JA and ABA in the treated leaves showed a downward trend (0–10 h), but these values were still higher than those of the control (Fig. 1G,H), except for the ABA content at 10 h. Moreover, we also determined the content of IAA (Fig. 1I). Surprisingly, the content of IAA in the cold acclimation stage (0 h) did not decrease, but under frozen stress (0 h–10 h), the content decreased significantly. In general, cold and freezing environments produce different hormonal responses.

### Analysis of sample Iso-Seq results

To identify and characterize the transcript dynamics of *M. sieversii* under freezing treatment after cold acclimation, we employed PacBio SMRT and RNA-seq technologies for transcriptome sequencing. In this study, the sample materials use for transcriptomic analysis and the treatment methods were consistent with the physiological measurements in Fig. 1. Subsequently, the cDNA libraries constructed from treated and CK samples were sequenced. After correcting the data, a total of 11,781,904 (20.79G) subreads were obtained (Table 1). In addition, a total of 611,407 circular consensus sequence (CCS) reads were obtained after filtering. Finally, 485,006 full-length non-chimeric reads (FLNC) were obtained and poly-A tails in which an average FLNC read length was 1765 bp. In total, 432,508,752 nucleotides were obtained, and 191,961 reads were generated. The minimum length was 193 bp, and the maximum length was 14,168 bp. The N50 value was 2613 bp, and the N90 value was 1344 bp. The length of each subread sequence was counted after quality control of the raw data, and the length distribution map was generated (Additional file 1).

**Table 1:** Transcript length distribution statistics

Sample	TML
Subreads base(G)	20.79
Subreads number	1,178,1904
Average subreads length	1765
CCS	611,407
5′-primer	579,181
3′-primer	579,874
Poly-A	518,402
Full length	488,828
FLNC	485,006
Average FLNC read length	2117
FLNC/CCS	0.79
Total_nucleotids	432,508,752
Total_number	19,1961
Mean_length	2254
Max_length	14,168
Min_length	193
N50	2613
N90	1344

TML represents mixed samples containing treated and control samples.

**Fig. 2:**
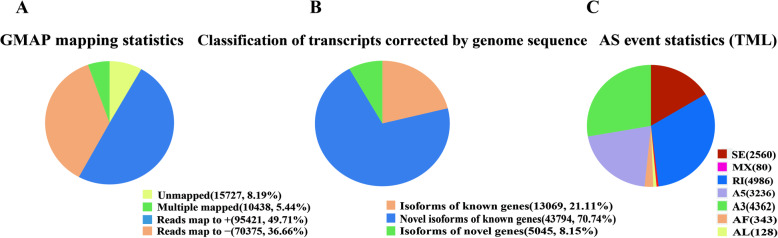
Characterization of *M. sieversii* transcripts. (A) GMAP mapping statistics. (B) Classification of transcripts corrected based on the reference genome sequences. (C) The gene numbers involving AS events. TML represents mixed samples containing treated and control samples

GMAP software was used to match the consensus sequences to the reference genome sequences, and a total of 176,234 (91.81%) reads were mapped to the *Malus domestica* genome sequences (GDDH13 v1.0); according to the results, the sequences can be divided into four types: unmapped, 15,727 (8.19%); multiple mapped, 10,438 (5.44%); reads mapped to the forward strand (+), 95,421 (49.71%); and reads mapped to the antisense strand (−), 70,375 (36.66%) (Fig. 2A). To obtain a comprehensive genetic annotation, the functions of novel genes were annotated based on seven databases. Of the 3917 novel genes, 3808 were significantly matched in at least one of the databases. Among them, 3359, 2242, 3216, 1625, 2038, 3708, and 2038 novel genes were found in the NR (NCBI nonredundant protein sequence), SwissProt, KEGG (Kyoto Encyclopedia of Genes and Genomes), KOG/COG (Clusters of Orthologous Groups of proteins), GO (Gene Ontology), NT (NCBI nucleotide sequences by BLAST, E-value 1e-10), and Pfam (Protein family) databases, respectively (Additional file 1). The histogram demonstrated that 1097 genes were simultaneously annotated across all seven databases. We compared the 61,908 transcripts with the *M. domestica* genome gene set, and they were classified into three groups, with following results are shown in Fig. 2B: isoforms of known genes (13,069, 21.11%), novel isoforms of known genes (43,794, 70.74%), and isoforms of novel genes (5045, 8.15%). Alternative splicing (AS) events were analyzed with SUPPA software. We detected 22,316 transcripts with alternative splicing (AS) events, including skipped exons (SE), mutually exclusive exons (MX), alternative 5′splice site (A5), alternative 3′splice site (A3), retained intron (RI), alternative first exon (AF) and alternative last exon (AL) (Fig. 2C, Additional file 6). The transcripts were analyzed by principal component analysis (PCA) (Additional file 2). The first two axes of the PCA represent the differentiation between the biological replicates of the different treatments. In addition, 1710 fusion transcripts were identified (Additional files 3, 7).

### Identification and analysis of DETs

**Fig. 3:**
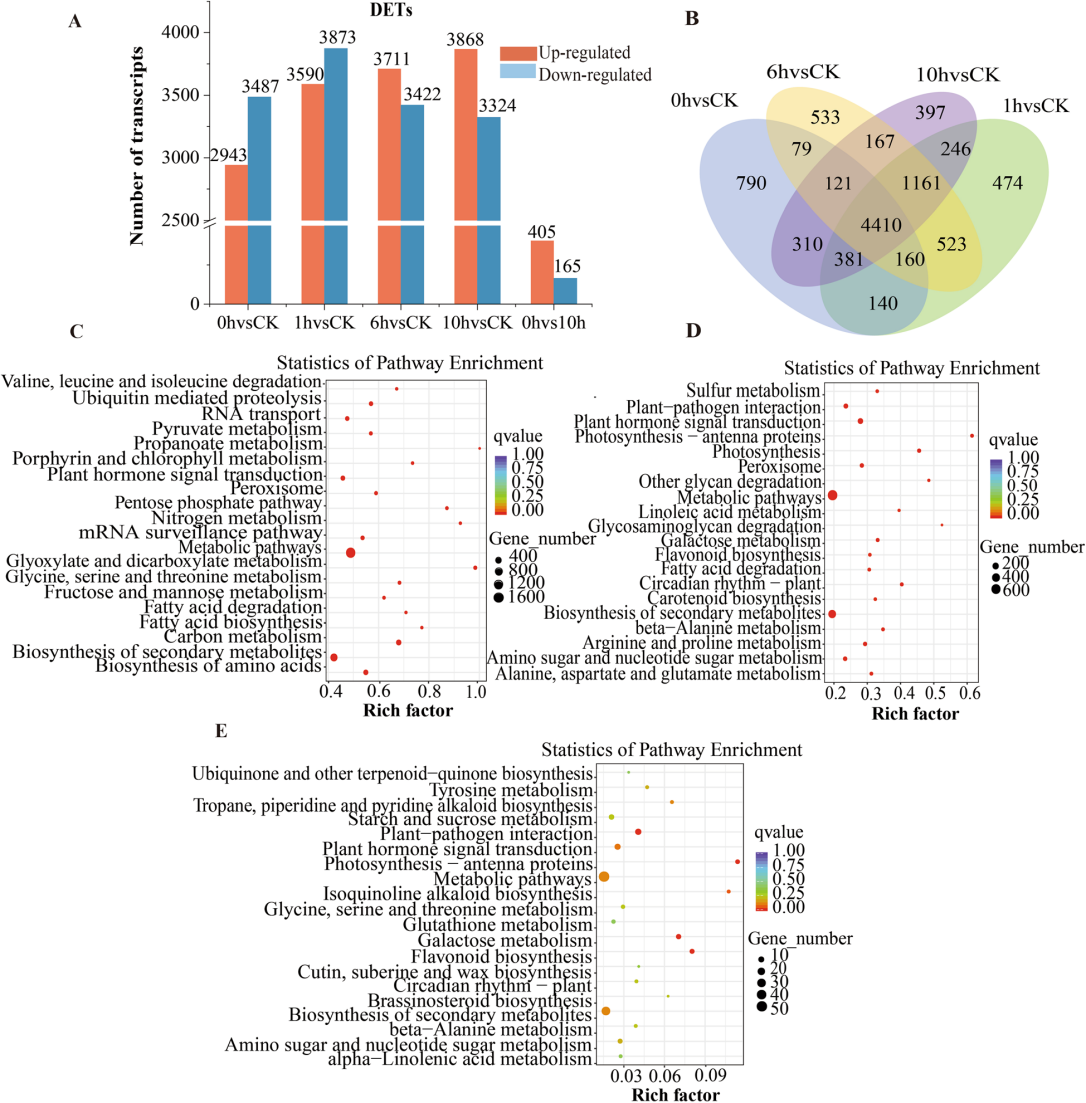
Analysis of differentially expressed transcripts (DETs) at different times (CK, 0, 1, 6, and 10 h) across the transcriptome*.* (A) Number of DETs in the pairwise analyses. Upregulated (orange) and downregulated (blue) transcripts were quantified. (B) Venn diagram of the DETs at the different time points compared with the CK; (C) KEGG pathway enrichment at 0 h vs CK; (D) KEGG pathway enrichment at 10 h vs CK; (E) KEGG pathway enrichment at 10 h vs 0 h. dpi. A rich factor was the ratio of an input number to the background number in a specific pathway. The size and color of the dots represent the transcript numbers and the q-values, respectively

In total, we obtained 24,716 DETs in different comparisons (Additional file 8). To better understand the gene expression changes between the CK and cold-treated apple leaf samples in response to freezing after 2 d of cold acclimation, we compared the number of DETs that were different among time points (0 h vs CK, 1 h vs CK, 6 h vs CK, 10 h vs CK, 10 h vs 0 h). A total of 6430 DETs were obtained between 0 h and CK, with 2493 upregulated and 3487 downregulated transcripts; additionally, 7463 (3590 upregulated and 3873 downregulated), 7133 (3711 upregulated and 3422 downregulated), 7192 (3868 upregulated and 3324 downregulated), and 570 (405 upregulated and 165 downregulated) transcripts were identified among the 1 h vs CK, 6 h vs CK, 10 h vs CK, 10 h vs 0 h comparisons, respectively (Fig. 3A). We used the Venn diagram to identify DETs that responded continuously during freezing stress. From the Venn results, a total of 2234 DEGs and 4410 DETs (2457 upregulated and 1953 downregulated) were shared across all treatments (Fig. 3B, Additional files 9, 10). We conducted a GO enrichment and KEGG pathway analysis on all upregulated and downregulated DETs to explore their biological functions (Additional files 11, 12, 13). In addition, KEGG pathway analyses were also conducted to identify several important pathways (Fig. 3C-E, Additional file 4,). In the pairwise, DETs were significantly enriched in the “biosynthesis of secondary metabolites”, “photosynthesis”, “peroxisome”, “starch and sucrose metabolites” and “plant hormone signal transduction” categories. All the pathways worked together to form a complex and efficient signaling network that reduced or even eliminated cold stress damage. The pathway enrichment results indicated that the major pathways included plant hormone signal transduction, starch and sucrose metabolism, peroxisomes, transcription factors, and photosynthesis.

### Sugar and starch metabolism pathway

**Fig. 4:**
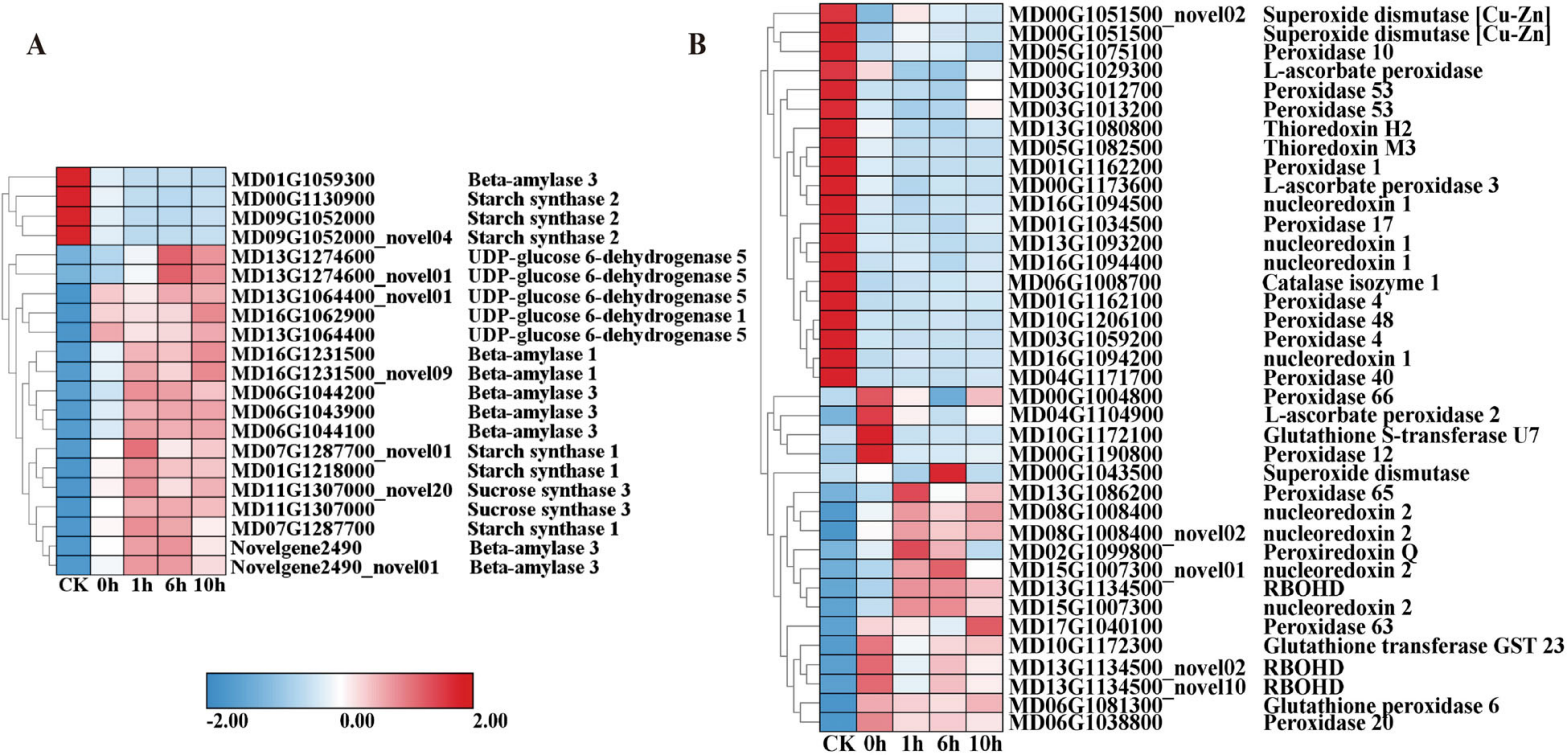
Heat map of the relative expression levels of DEGs involved in sugar metabolism and the antioxidant defense system; (A) Sugar and starch metabolism-related genes; (B) Antioxidant-related genes. The red and blue colors indicate high and low expression levels (log2 fragments per kilobase of transcript per million mapped reads, FPKM), respectively

The sugar and starch metabolism pathway (Fig. 4A) showed that two related starch synthase 2 (*SS2*) genes (MD09G1052000 and MD00G1130900) were downregulated. However, another *SS1* gene (MD01G12218000) was upregulated. Sucrose synthase 3 (*SUS3*) gene (MD11G1307000, MD11G1307000_novel20) was upregulated. Notably, the gene expression of the Bate-amylase (BAM) family members (MD16G1231500, MD16G1231500_novel09, MD06G1044200, MD06G1043900, Novelgene2490, Novelgene2490_novel01, MD07G1287700, and MD06G1044100) was significantly upregulated under freezing stress. It is worth noting that the UDP glucose 6-dehydrogenase genes (*UGDH*) (MD16G1062900, MD13G1064400, MD13G1064400_novel01), which catalyze UDP-alpha-D-glucuronate, exhibited inducible expression. In short, there are up and downregulated genes across various biological reactions that may occur in wild apple plants in response to cold stress.

### Antioxidant defense system

According to the heat map of the antioxidant-related gene expression levels of DEGs (Fig. 4B), superoxide dismutase 1 (*SOD1*) (MD00G1051500, MD00G1051500_novel02), L-ascorbate peroxidase T (*APXT*) (MD00G1029300), peroxidase (*POD*) (MD05G1075100, MD03G1012700, MD10G1206100, MD04G1171700, MD03G1059200, MD01G1162100), thioredoxin (*TRX*) (MD05G1082500, MD13G1080800), respiratory burst oxidase homolog D (*RBOHD*) (MD13G1134500, MD13G1134500_novel02, MD13G1134500_novel10), and nucleoredoxin (*NRX*) (MD13G1093200, MD16G1094400, MD16G1094400) were downregulated. However, glutathione S-transferase U7 (*GSTU7*) (MD10G1172100), *PER* (MD17G1040100, MD13G1134500, and MD13G1086200), glutathione transferase 23 (*GST23*) (MD10G1172300), and catalase isozyme1 (*CAT1*) (MD06G1008700) genes were significantly upregulated under freezing stress conditions.

### Responses of the plant hormone signaling network to cold stress

**Fig. 5:**
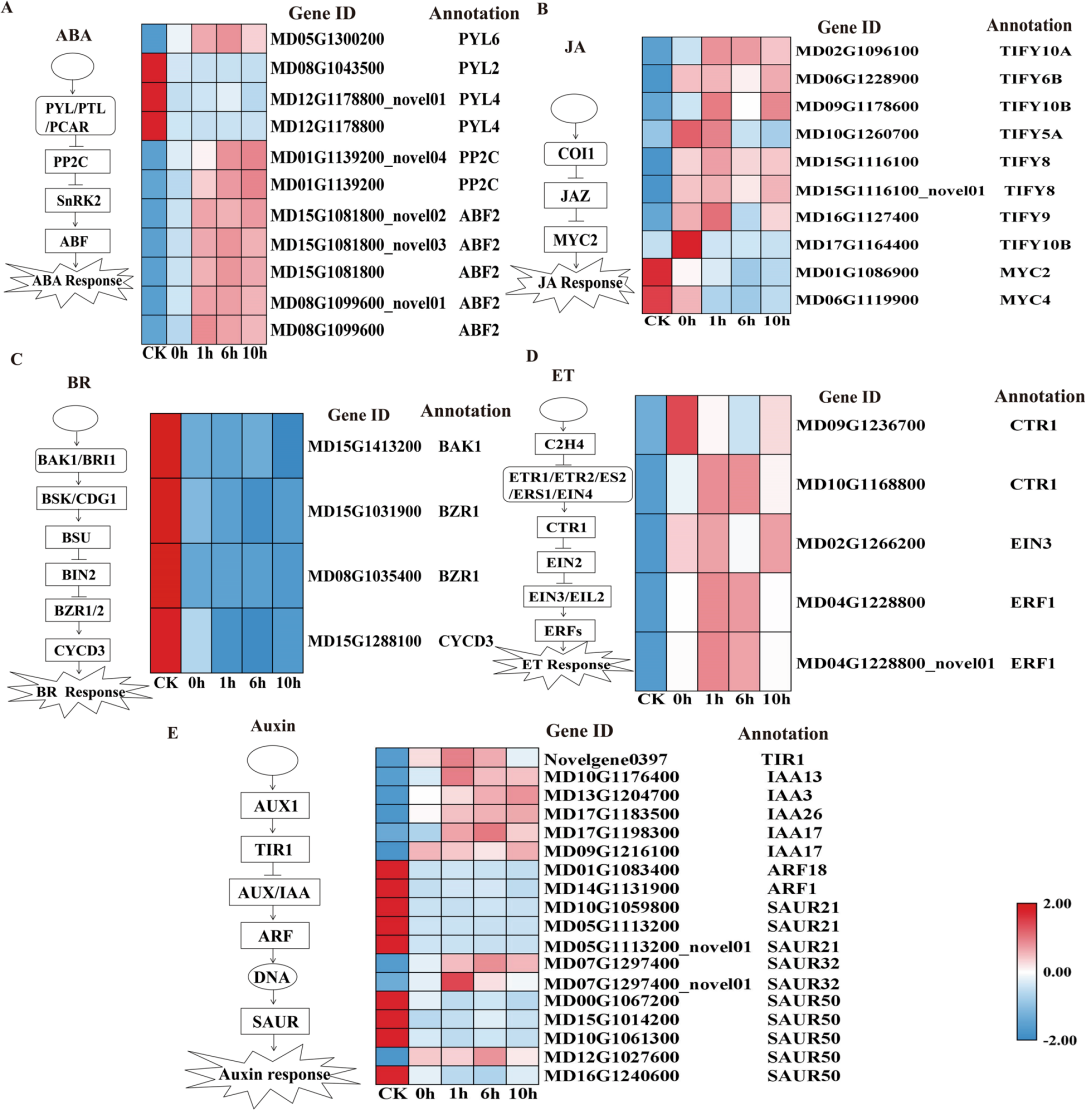
Plant signal transduction pathways and expression of related genes in *M. sieversii*. (A) Heatmap of the ABA signal transduction pathway. (B) Heatmap of the JA signal transduction pathway. (C) Heatmap of the BR signal transduction pathway. (D) Heatmap of the ET signal transduction pathway. (E) Heatmap of the auxin signal transduction pathway. The red and blue colors indicate high and low expression levels (log2 fragments per kilobase of transcript per million mapped reads, FPKM), respectively

In the ABA signal transduction pathway (Fig. 5A), 2 *PYL* genes (MD08G1043500, MD12G1178800, MD12G1178800_novel01) were upregulated under freezing stress conditions, whereas another *PYL* gene (MD05G1300200), a protein phosphatase 2C (*PP2C*) gene (MD01G1139200, MD01G1139200_novel04), and two *ABF* genes (MD15G1081800*,* MD15G1081800_novel02, MD15G1081800_novel03, MD08G1099600, MD08G1099600_novel01) were downregulated. In the JA signal transduction pathway (Fig. 5B), several TIFY proteins (MD02G1096100, MD06G1228900, MD09G1178600, MD10G1260700, MD15G1116100, MD15G1116100_novel01, MD16G112740, and MD17G1164400) were induced under freezing stress in this study. In the BR signal transduction pathway (Fig. 5C), BRASSINAZOLE RESISTANT1 (*BZR1*) (MD15G1031900, MD08G1035400), BRI1-associated kinase 1 (*BAK1*) (MD04G1054400), and Cyclin D3 (*CYCD3*) (MD15G1288100) were downregulated. In the phytohormone ethylene (ET) signal transduction pathway (Fig. 5D), serine/threonine-protein kinase 1 (*CTR1*) (MD09G1236700, MD10G1168800) and ETHYLENE INSENSITIVE 3 (*EIN3*) (MD02G1266200), and ethylene-responsive transcription factor (*ERF1*) (MD04G1228800, MD04G1228800_novel01) were significantly upregulated under freezing stress. In the auxin signal transduction pathway (Fig. 5E), TRANSPORT INHIBITOR RESPONSE (*TIR*) (Novelgene0397), IAA/indole/− 3-acetic acid (*AUX/IAA*) (MD10G1176400, MD13G1204700, MD17G1183500, MD17G1198300, MD09G1216100), and small-IAA-up-RNA (*SAUR*) (MD07G1297400, MD07G1297400_novel01, MD16G1240600) were upregulated, whereas *ARF* (MD01G1083400, MD14G1131900) and *SAUR* (MD10G1059800, MD05G1113200, MD05G1113200_novel01, MD00G1067200, MD15G1014200, MD10G1061300, MD16G1240600) were downregulated.

### Photosynthesis

The KEGG enrichment analyses of DETs showed that the photosynthesis pathway was significantly enriched. We focused our attention on photosynthesis-related DETs and found that most of these transcripts were downregulated under cold conditions. In PSI, the DEGs photosystem I light reaction center (MD01G1004300, MD17G1191200, MD17G1191400, MD13G1191300, MD03G1042800, MD10G1238800, MD16G1191400), photosystem I subunit O *(PsaO)* (MD03G1150500, MD03G1150500_novel01), and ATP synthase subunits (*ATPase*) (MD05G1347300, MD17G1003000) were downregulated. In PSII, the DEGs oxygen-evolving enhancer protein 2 (*OEEP2*) (MD17G1132900, MD07G1 190900, MD07G1191200, MD09G1146300), oxygen-evolving enhancer protein 3 (*OEEP3*) (MD10G1311700, MD11G1227900), PSII light reaction center (MD10G1176000), *PsbW* gene (MD10G1192100), and *PsbY* genes (MD16G1106800, MD16G1106800_novel01, MD13G1106700, MD13G1106700_novel01) were downregulated. However, two of the DEGs related to photosynthesis were upregulated, PSII core complex gene *psbY* (MD06G1160500) and PSII reaction center gene *PSB28* (MD10G1176000), during low-temperature stress in *M. sieversii*. The results are shown in Table 2.

**Table 2:** Freezing-responsive DETs involved in photosynthesis in *M. Seversii*

KEGG ID	Gene_ID	Annotation	Log_2_FC 0 h vs CK	Log_2_FC 1 h vs CK	Log_2_FC 6 h vs CK	Log_2_FC 10 h vs CK
K02701	MD01G1004300	photosystem I reaction center subunit N	−2.4191	−1.8425	− 1.6728	− 1.6678
K02695	MD17G1191200	photosystem I reaction center subunit VI	−2.5274	−2.2878	− 2.1296	−2.0659
K02695	MD09G1209500	photosystem I reaction center subunit VI	−1.6946	− 1.5407	− 1.3918	−1.2528
K02695	MD17G1191400	photosystem I reaction center subunit VI	−2.1974	− 2.0681	− 1.9622	−1.9178
K02692	MD13G1191300	photosystem I reaction center subunit II	−1.7756	− 1.7049	− 1.508	−1.4809
K08905	MD03G1042800	photosystem I reaction center subunit V	−1.7683	−1.5376	− 1.3845	−1.2196
K02694	MD10G1238800	photosystem I reaction center subunit III	−2.0254	−1.8472	− 1.6623	− 1.4185
K02692	MD16G1191400	photosystem I reaction center subunit II	−2.401	− 2.349	− 2.0606	−1.9346
K02694	MD05G1259200	photosystem I reaction center subunit III	−1.3697	−1.4181	− 1.3319	−1.1084
K02698	MD05G1276100	photosystem I reaction center subunit psaK	−2.9286	−3.6163	− 3.1435	− 2.9163
K02641	MD02G1130800	ferredoxin	−1.9304	− 1.7868	− 1.7782	−1.5045
K02641	MD02G1130800_novel01	ferredoxin	−1.83335	− 1.77998	− 1.72846	−1.83335
K14332	MD03G1150500	photosystem I subunit O	−2.0745	−1.9282	−1.6151	− 1.5103
K14332	MD03G1150500_novel01	photosystem I subunit O	−1.889	− 1.81609	− 1.56587	− 1.43579
K02115	MD05G1347300	ATP synthase gamma chain	−1.1495	−1.232	− 1.2582	−1.0038
K02113	MD17G1003000	ATP synthase subunit delta	−1.5733	−1.6869	− 1.5763	−1.5271
K02723	MD06G1160500	photosystem II core complex proteins psbY	3.8231	3.6315	3.5436	3.5162
K02717	MD17G1132900	photosystem II oxygen-evolving enhancer protein 2	−1.6332	−1.4713	−1.3362	− 1.0575
K02717	MD07G1190900	photosystem II oxygen-evolving enhancer protein 2	−1.1908	−1.4006	− 1.2204	−1.1741
K02717	MD07G1191200	photosystem II oxygen-evolving enhancer protein 2	−1.0559	−1.4264	− 1.5194	−1.1525
K08901	MD10G1311700	photosystem II oxygen-evolving enhancer protein 3	−2.6605	−2.2329	−1.9441	−1.5594
K08901	MD11G1227900	photosystem II oxygen-evolving enhancer protein 3	−1.5407	−2.1963	−2.0574	− 2.0248
K02717	MD09G1146300	photosystem II oxygen-evolving enhancer protein 2	−1.9933	−1.695	− 1.4987	−1.2512
K08903	MD10G1176000	photosystem II reaction center PSB28 protein	1.3279	1.131	1.0186	1.2959
K02721	MD10G1192100	photosystem II PsbW protein	−1.5838	−1.391	−1.1383	− 1.2135
K02723	MD16G1106800	photosystem II PsbY protein	−2.5193	−3.2396	−2.8513	− 2.5848
K02723	MD16G1106800_novel01	photosystem II PsbY protein	−2.46007	−3.08437	−2.69523	− 2.62407
K02723	MD13G1106700	photosystem II PsbY protein	−2.2263	−2.213	−1.893	−1.9343
K02723	MD13G1106700_novel01	photosystem II PsbY protein	−2.15847	−2.15399	−1.832	−1.95485
K02636	MD09G1098300	cytochrome b6-f complex iron-sulfur subunit	−2.1886	−2.0483	− 2.0024	−1.8187
K02636	MD09G1098300_novel02	cytochrome b6-f complex iron-sulfur subunit	−2.30428	−2.11278	− 2.15924	−2.02768
K02638	MD15G1006300	plastocyanin	−1.6645	−1.5692	− 1.3261	−1.1126

### Transcription factors (TF)

**Fig. 6:**
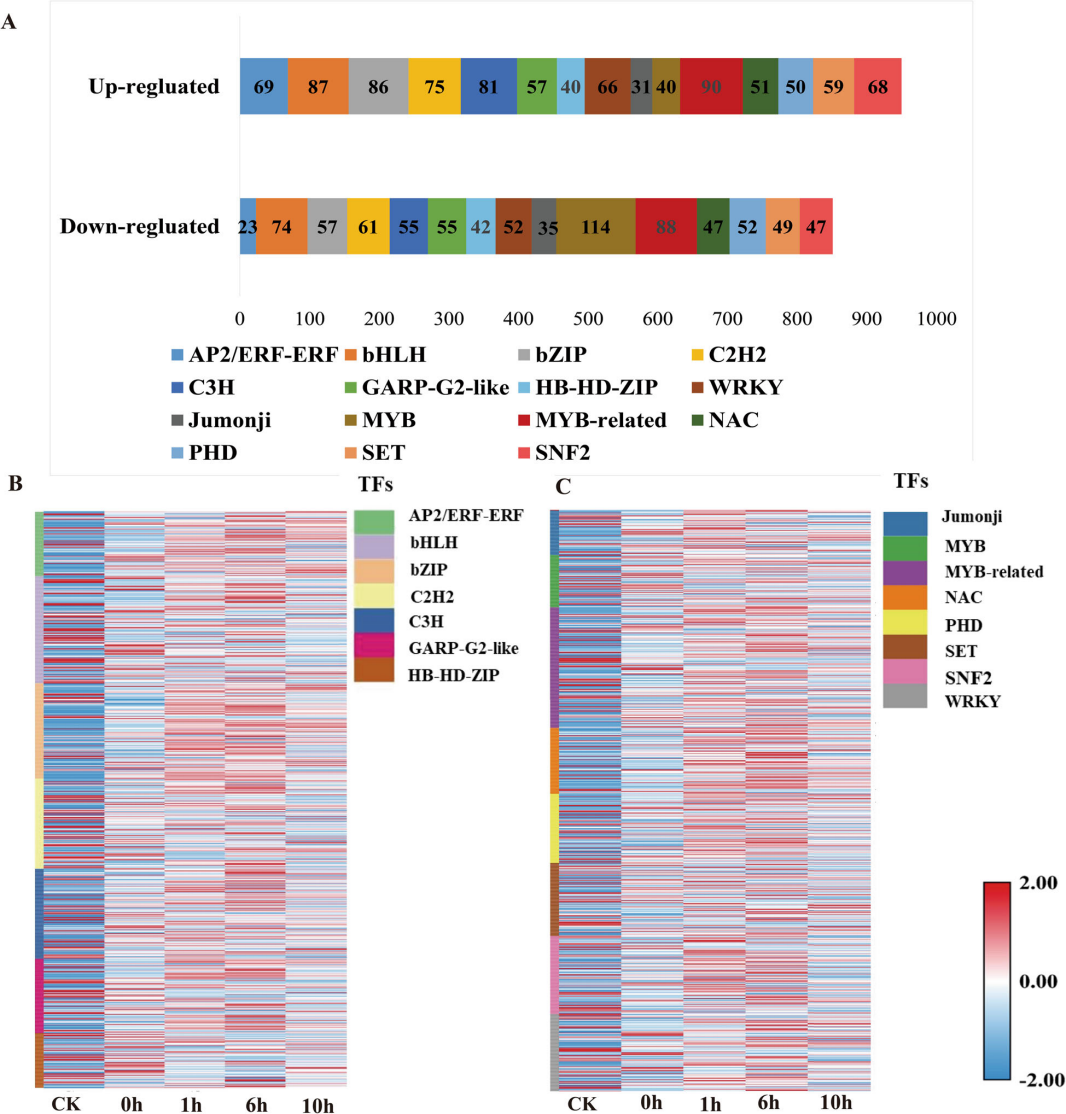
Identification and analysis of the transcript expression levels of the top 15 transcription factor families. (A) Statistics of the differentially expressed transcription factors. (B) Expression profiles of differentially expressed TFs among different samples. The heat map was generated from the FPKM values. The red and blue colors indicate high and low expression levels, respectively

TFs are known to play essential roles in plant responses to abiotic stress. The iTAK and HMMER analysis of apple transcripts allowed the identification of nonredundant TFs. In our work, we analyzed the expression patterns of 1801 transcripts of 676 genes in the top 15 transcription factor families (Fig. 6, Additional file 14). Heatmaps of abundance-altered TFs were plotted to depict the transcript abundance profiles of each TF family (Fig. 6B, C). The MYB superfamily was the largest, including 154 MYB and 178 MYB-related members. The second-largest family was the bHLH family, with 161 members, followed by the bZIP (143), C2H2 (136), C3H (136), WRKY (118), SNF2 (112), GARP-G2-like (112), PHD (102), NAC (98), AP2-ERF (92), SET (108), HB-HD-ZIP (82), and Jumonji (66) families.

### Validation of DETs by qRT-PCR analysis

Sixteen DETs related to cold adaptation in different signaling pathways and TFs were randomly selected to validate the gene expression levels using qRT-PCR. They represented various functional categories or pathways, including sugar metabolism (*SS1*, *SUS3*), antioxidant defense systems (*CAT1, GPX6*), plant hormone signal transduction (*PP2C, ABF2, ARF9, IAA17*), TFs (*NAC104, ERF3*), and photosynthesis systems (*psaD*, *PSB27,* Cyt *b6-f*). Nine DETs showed elevated expression levels, and the expression levels of seven DETs decreased. The results show that the expression patterns were clearly consistent between qRT-PCR and transcriptome sequencing (Additional file 5). The independent qRT-PCR results confirmed the accuracy and reliability of the RNA-seq results.

## Discussion

Apple is one of the most important fruit crops worldwide. *M. sieversii*, a progenitor of domesticated apple, represents a reservoir of genetic diversity and is widely used as a rootstock in China [28]. The selection of rootstocks is usually based on stress tolerance [29]. Cold stress is one of the most severe abiotic stresses in apple plants and is one of the many limiting factors for global apple production. Through cold acclimation, plants can alleviate the harm of low temperatures and enhance their tolerance to cold stress [30]. In tea plants, differential changes in transcription and metabolism levels occurred in response to CA and cold stress conditions [31]. Since the cold-stress response network is very complex and contains an array of biochemical and physiological modifications, many defense mechanisms are activated during cold acclimation, thus the transcriptome changes will be less intense even when the plant is exposed to lower temperatures. To discover the key functional and regulatory genes of cold resistance in *M. sieversii*, we examined seedlings under cold stress after 2 d of cold acclimation and performed full-sequence transcriptomic analysis. Full-sequence transcriptomic data have great potential for discovering new or previously unrecognized isoforms, meanwhile, different expression patterns appearing between isoforms of the same gene. For example, in present work, isoforms MD05G1126500_novel01, MD08G1162500_novel01, MD12G1040700_novel07, MD12G1126100_novel02, MD05G1126500_novel01, MD06G1228000 are up-regulated, another isoforms of the corresponding gene MD05G1126500_novel02, MD08G1162500, MD12G1040700_novel06, MD12G1126100_novel08, MD05G1126500_novel02, MD06G1228800_novel01 are down-regulated. In addition, the transcriptome results revealed that isoforms involved in transcription regulation, signal transduction of hormones, transcription factors, and oxidative stress were differentially expressed under cold stress, we also compared the activities of MDA and Fv/Fm and the contents of ABA and JA in seedlings of *M. sieversii.* To further explore the response mechanism of apple under freezing stress, we identified a series of genes from different pathways. This study provides a novel understanding of the roles of CA in freezing stress in *M. sieversii*.

### Sugar and starch metabolism

The metabolism of sugar and starch plays a vital role in the cold resistance of plants. Sugar can be used as a signaling molecule in hormone synthesis, plant growth and development, and various antistress reactions, as a nutrient to supply energy and as a protective agent to avoid cold damage [32, 33]. SUS is a key enzyme involved in sucrose metabolism and is part of a family of genes encoding different sucrose isozymes, including *SUS1* and *SUS3* (MD11G1307000, MD02G1100500) were upregulated. Here, the induction of two genes encoding SUS may point towards the degradation of sucrose to UDP-glucose and fructose. *UGDH* (MD16G1062900, MD13G1064400) was induced. Under low-temperature stress, the task of increasing glycolysis can be completed [34]. Starch hydrolysis was associated with increased maltose accumulation and increased expression of BAM, which encodes the beta-amylase necessary for starch mobilization and raises the levels of soluble sugars capable of acting as osmolytes or antioxidants [35–37]. Under cold stress conditions, the activity of *BAM1* was largely unaffected, but *BAM3* expression increased [38]. In this study, two related *SS* genes (MD09G1052000 and MD00G1130900) were repressed, and another *SS1* (MD01G1218000) was induced. Notably, the gene expression of BAM family members, such as MD16G1231500, MD06G1044200, MD06G1043900, and MD06G1044100, was significantly upregulated under freezing stress (Fig. 4A). This phenomenon may indicate that Xinjiang wild apples respond to freezing stress by accelerating the decomposition of starch to provide energy and achieve heat preservation.

### Antioxidant defense system

In the antioxidant defense system, various nonenzymatic and enzymatic ROS detoxification mechanisms have developed in plant cells. Major enzymatic systems include superoxide dismutase (SOD), ascorbate peroxidase (APX), peroxidase (POD), and catalase (CAT) [39]. MDA levels and electrolyte permeability are commonly used as indicators of membrane structural damage and plant resilience [40, 41]. Under abiotic stress, antioxidant enzymes can eliminate harmful substances to the greatest extent and effectively reduce membrane damage. Plants with suppressed APX production induce SOD and CAT to compensate for the loss of APX, whereas plants with suppressed CAT production induce APX and GPX. In addition, POD and CAT are hydrogen peroxide scavengers and catalyze the conversion of hydrogen peroxide (H_2_O_2_) into water. In this study, genes encoding *CAT* (MD06G1008700), *GST* (MD10G1172300), *GP*X6 (MD06G1081300), *APXT* (MD04G1104900), *SPS1* (MD10G1258500) and *RBOHD* (MD13G1134500, MD13G1134500_novel02, MD13G1134500_novel10) were upregulated under freezing stress conditions. However, the genes encoding *POD* (MD03G1013200, MD10G1206100, MD04G1171700, MD03G1059200, and MD01G1162100) were significantly downregulated (Fig. 4B). Overall, the antioxidant defense system-related genes also rapidly responded to freezing stress in Xinjiang wild apples, enabling plants to cope with freezing stress. Antioxidant defense systems involve many kinds of antioxidant genes, which have unique functions under stress conditions, forming a complex antioxidant defense system in vivo.

### Responses of the plant hormone signaling network to cold stress

We measured ABA accumulation at − 4 °C for different times after cold acclimation. When cold acclimation ended, a large amount of ABA accumulated. ABA levels were increased in plants that were stressed, such as those under drought, cold, or high temperature stresses, and ABA played a crucial role in plant stress resistance [42–44]. During cold acclimation, ABA accumulation can induce the expression of some cold-resistant genes to improve freezing resistance [45]. Numerous studies found a direct link between the accumulation of ABA in woody plants and the increase in cold tolerance [46]. Important components of ABA include *PP2C* and *PYL*, which is closely related to the level of plant stress resistance [47, 48]. *PYL* (MD05G1300200) and *PP2C* (MD01G1139200, MD01G1139200_novel04) were induced in response to cold stress. *ABF2* regulates various aspects of the ABA response by controlling the expression of a subset of ABA-responsive genes [49, 50]. In this study, *ABF* (MD08G1099600, MD08G1099600_novel01, MD15G1081800, MD15G1081800_novel02, MD15G1081800_novel03) was upregulated under freezing stress conditions. These results indicated that ABA stimulated this signaling pathway and that DEGs were involved in the ABA signaling response to freezing stress.

We also measured the content of JA, which show a consistent trend as that for ABA content. After cold acclimation (0 h), the content of JA increased significantly, while the content of JA decreased during the freezing treatment (0 h–10 h). However, the JA content was still higher than that of the control. Similar to ABA, cold stress induces a number of JA biosynthetic and signaling genes that ultimately trigger the accumulation of JA and increase cold tolerance by regulating the ICE-CBF/DREB1 cascade [51]. Consistent with the positive role of JA in freezing stress, the production of endogenous JA was triggered by cold treatment. Exogenous application of MeJA significantly improved plant freezing tolerance with or without cold acclimation [52]. In JA signal transduction pathway, *JAZ* genes (MD02G1096100, MD06G1228900, MD09G1178600, MD10G1260700, MD15G1116100, MD15G1116100_novel01, MD16G112740, and MD17G1164400) was upregulated under freezing stress in this study. The bHLH transcription factor *MYC2* (MD01G1086900, MD06G1119900, and MD11G1254100) acted as a direct target of the JAZ protein to prevent JA signaling. In this study, JAZ protein was induced under freezing stress, whereas the transcription factor *MYC2* was repressed. Taken together, these results indicated that the JA signaling pathway regulated Xinjiang wild apple freezing tolerance after cold acclimation.

BR and ET also play major roles in plant defense responses to cold stress [53]. It is worth noting that in BR signal transduction pathways, *BAK1* (MD04G1054400), a transmembrane receptor kinase; *BZR1* (MD15G1031900, MD08G1035400), a core member of the BR signaling pathway; and *CYCD3* (MD15G1288100) were repressed under cold stress (Fig. 5). ET levels increase in response to cold stress and can induce the accumulation of antifreeze proteins [54]. In this study, genes encoding *CTR1* (MD09G1236700, MD10G1168800), *EIN3* (MD02G1266200), and *ERF1* (MD04G1228800, MD04G1228800_novel01) were upregulated. In the ET signaling pathway, *CTR1* is a positive regulator in the ethylene signaling pathway. *EIN3* is controlled by a ubiquitin proteasome system, and *ERF1* is an immediate target for *EIN3* [55]. The results indicated that these genes in the ethylene signaling transduction pathway responded to freezing stress. These results indicate that both the BR and ET signaling pathways may function in *M. sieversii* cold response processes after acclimation.

In particular, the IAA content did not decrease significantly during cold acclimation but decreased significantly during freezing stress. We demonstrated that the endogenous hormone IAA may have a special role in cold acclimation. Moreover, the decreasing trend of the IAA content under freezing stress is similar to that of ABA and JA, which may involve coordination with ABA and JA. Although the role of auxin after cold stress is not clear, genes related to the IAA signal transduction pathway were differentially expressed in this study. *IAA* (MD10G1176400, MD13G1204700) and *SAUR* (MD12G1027600, MD07G1297400, MD07G1297400_novel01) were induced in response to low temperature (Fig. 5E), whereas *ARF* (MD01G1083400, MD14G1131900) and *SAUR* (MD10G1059800, MD10G1061300) were repressed. Analysis of genes related to the IAA signal transduction pathway showed that the *TIR1* (Novelgene0397) transcript was highly expressed. *TIR1* can cause AUX/IAA ubiquitination, relieving the inhibition of ARF activity by AUX/IAA and hence initiating the transcription of IAA-induced genes. Recent studies have shown that plant changes induced by cold stress are closely related to the auxin response [56–58]. These genes were differentially expressed in response to low temperature, indicating that the auxin signaling pathway may participate in the *M. sieversii* cold response. In conclusion, the *M. sieversii* freezing response after CA involves a complicated regulatory network containing multiple types of plant hormone signaling pathways.

### Photosynthesis

The direct impact of abiotic stress on the activity of photosynthesis was the disruption of all photosynthetic components, such as photosystems I and II, electron transport, carbon fixation, ATP generation, and stomatal conductance [59], which resulted in serious damage to the photosynthetic machinery of plants [60]. In this study, DEGs associated with photosynthesis showed that most of these genes were downregulated, such as *PsaN* (MD01G1004300), *PsaL* (MD04G1244400, Novelgene0781), *PsaO* (Novelgene3166), *PsbP* (MD17G1132900, MD09G1146300), *PetC* (MD09G1098300, MD09G1098300_novel02), and *ATPc* (MD05G1347300). These results suggest that photosynthesis may be inhibited by freezing conditions. It is worth noting that two genes were upregulated, PSII core complex gene *psbY* (MD06G1160500) and PSII reaction center gene *PSB28* (MD10G1176000). The evidence of genes that encode proteins linked to photosynthesis in the previous study as *psbY* is indicating that the slowdown in the degradation of chlorophylls by 1-MCP would also affect other photosystem II-related proteins [61]. *PsbY* gene up-regulation expression may play a critical role in photosynthetic systems under freezing stress. Low temperatures also cause a steady-state light imbalance, which reduces the quantum efficiency of PSII. This implies that freezing stress could induce a series of changes in genes related to photosynthetic pathways, thus decreasing Fv/Fm values.

### Transcription factor dynamics during freezing responses

In this study, 1801 transcripts of 676 TFs from the top 15 TF families responding to freezing stress were examined (Fig. 6A), and the expression patterns of the top 15 TF families were analyzed (Fig. 6B,C). Many of these families of TFs were reported to play central roles in plant responses to abiotic stress, including cold stress [62–66]. A total of 49 AP2/ERF members were detected under freezing stress. *DREB1/CBF* (MD04G1067800, MD04G1067800_novel01, MD04G1067800_novel02, MD04G1165400, MD04G1165400_novel01), a subfamily member of AP2/ERF, was identified to be induced at least tenfold and twofold under freezing stress, respectively. Similarly, in previous studies, the expression of the CBF gene was upregulated in apple bark at different freezing temperatures [20]. Similarly, *MYB113* (MD09G126510), *MYB1* (MD09G1278600, MD09G1278600_novel02), *MYB62* (MD13G1039900), *MYB108* (MD13G1148200, MD13G1148200_novel01) and *MYB4* (MD16G1218900) strongly responded to freezing stress after cold acclimation, and this response continued throughout the whole freezing stress period. In previous studies, three MYB transcription factors (*MdMYB12, MdMYB22,* and *MdMYB114*), which had several CBF/DREB response elements in their promoters, were significantly induced by low-temperature exposure and their expression also correlated highly with anthocyanin accumulation [11]. In apple, MYB transcription factors, such as *MYB1* and *MYB113, MYB114* are involved in the regulation of anthocyanin synthesis pathways [67, 68]. During freezing stress, low temperature induces the expression of MYB transcription factors and enhances anthocyanin synthesis to enhance freezing resistance. In addition, recent investigations in apple show that *MdMYB4-*transgenic apple calli exhibited enhanced tolerance to cold stress, as indicated by reduced electrolyte leakage value, and MDA content than the control calli [69]. The upregulation of *MYB4* may increase cold resistance in wild apples. It also appears likely that WRKY TFs play important roles in *M. sieversii* cold regulation. Under freezing stress, 118 WRKY members were differentially expressed, among which *WRKY71* (MD10G1266400, MD10G1266400_novel01), *WRKY6* (MD10G1324500), and *WRKY70* (MD12G1189900) were induced at least 5-fold. In *Arabidopsis*, WRKY70 in promoting BR-mediated gene expression to regulated plant growth and drought response, WRKY71 activity hastens flowering, thereby providing a means for the plant to complete its life cycle in the presence of salt stress [70, 71]. In wild apple, the high expression level of WRKY70 and WRKY71 may affect plant growth to cope with low temperature environment. Our transcriptome data also showed that other transcription factors, such as bHLH (161), bZIP (143), C2H2 (136), C3H (136), WRKY (118), SNF2 (112), GARP-G2-like (112), PHD (102), NAC (98), AP2-ERF (92), SET (108), HB-HD-ZIP (82), and Jumonji (66) families, were induced. These important abiotic stress response regulators may function under cold stress conditions in *M. sieversii,* providing important genetic resources for cold-tolerant domesticated apple breeding.

## Conclusion

In this study, we first present the transcriptome of *M. sieversii* using PacBio single-molecule long-read sequencing to analysis of the responses to freezing temperature after cold acclimation. The expression patterns of 4410 DETs were determined, and identify several important pathways genes such as genes encoding superoxide dismutase, sucrose synthase, plant hormone-signaling transduction kinase, and peroxidase. In addition, a total of 1801 transcripts were confirmed as TFs. Some different members of the AP2/ERF, WRKY, MYB, and bHLH gene families were significantly up/downregulated. Furthermore, the transcription factors *DREB1/CBF, MYC2, WRKY71, WRKY6, WRKY70, MYB4* and *MYB88,* were strongly induced during the whole stress period. In short, cold acclimation can induce the continuous expression of some antifreeze genes during freezing stress. These findings provide a characterization of gene transcription and facilitate the understanding of the mechanisms of tolerance to low temperature after cold acclimation in *M. sieversii*.

## Methods

### Plant materials

Seeds of *M. sieversii* were collected in September 2016 from one area (43°23′2.20″N; 83°35′43.48″E) in a natural wild reserve forest in Yili, Xinjiang. With the permission of the Xinyuan County Forestry Bureau, seeds were obtained from the field and identified [72]. The field investigation and sample collection experiments were performed under institutional guidelines in accordance with local legislation. The germplasm of *M. sieversii* was identified by Ph.D. Wenjun Li, who worked at the Xinjiang Institute of Ecology and Geography, Chinese Academy of Sciences. A voucher specimen was deposited in the Natural Museum, Xinjiang Institute of Ecology and Geography, Chinese Academy of Sciences. (MS-20160919-015). *M. sieversii* seedlings were planted in 1/2 Murashige and Skoog (MS) medium with 30 g·L^− 1^ sucrose and 6 g·L^− 1^ agar in a greenhouse with 32 μmol·m^− 2^ s^− 1^ light intensity, 40–50% relative humidity, and a 16 h light/8 h dark photoperiod at 24 °C. Eight-week-old well-developed wild apples grew in medium at 4 °C under a 16 h photoperiod for two days (cold acclimation). Then, the chamber was cooled gradually (2 °C/h) from 4 °C to − 4 °C for 4 h (Boxun, LRX-580C-LED). Remove upper and lower three leaves and the middle leaves were separately harvested at the time points of CK, 0, 1, 6 and 10 h and each sample contained three biological replicates. Leaf samples were frozen in liquid nitrogen and stored at − 80 °C before physiological change analysis, hormone concentration determination, and RNA extraction. The RNA-seq was conducted using fifteen samples (CK, 0, 1, 6, 10 h) and the PacBio sequencing was implemented using the mixture of the samples.

### Measurement of physiological changes and endogenous hormone contents

Before sequencing the transcriptome, we determined the physiological indicators. MDA was measured according to a previously published method [73]. Chlorophyll fluorescence was recorded with a PAM2500 fluorometer (Walz, Germany), and the maximal quantum efficiency of PSII was calculated as Fv/Fm (Fv = Fm-F0). REL was conducted using the procedure described by this method [74], and it was calculated based on the following equation:

$$ \mathrm{REL}\ \left(\%\right)=\left[\left(\mathrm{EC}1-\mathrm{EC}0\right)/\left(\mathrm{EC}2-\mathrm{EC}0\right)\right]\times 100\%. $$

The determination of endogenous ABA and JA content was performed using HPLC (Comin Suzhou, China), with three replicates for each analysis.

### RNA quantification and transcriptome sequencing

Total RNA was isolated from the leaves of freeze-treated and untreated *M. sieversii* samples using the E.Z.N.A. Plant RNA Kit (Omega Bio-Tek, Norcross, GA, USA). Equal quantities of RNA from three biological replications at each stage were pooled to construct a complementary cDNA library. RNA concentration and quality were measured using a NanoDrop 2000 spectrophotometer (Thermo Fisher Scientific, Waltham, Massachusetts, USA). cDNA library construction was performed by high-throughput sequencing using the PacBio and Illumina platforms at Novogene (Beijing, China). Additional nucleotide errors in consensus reads were corrected using the RNA-seq data with the software LoRDEC [75]. Index of the reference genome sequences was built using HISAT2 (v2.1.0) and paired-end clean reads were aligned to the reference genome sequences using HISAT2. Consensus reads were aligned to the reference genomic sequence (GDDH13, Version 1.0) using GMAP with parameters-no-chimeras-cross-species-expand-off sets1-B5-K50000-f samse-n1 against the reference genome sequences [76]. Gene structure analysis was performed using the TAPIS pipeline [77]. The GMAP output bam format file and gff/gtf format genome sequences annotation file were used for gene and transcript determination. Alternative splicing events and alternative polyadenylation events were then analyzed. Fusion transcripts were determined as transcripts mapped to two or more long-distance genes and were validated by at least two Illumina reads.

### Iso-Seq data analysis

Raw reads were processed into error-corrected reads of interest (ROIs) according to the conditions full passes ≥0 and sequence accuracy> 0.75. By detecting whether each ROI sequence contains a 5′ primer, 3′ primer, and poly-A tail, the sequences can be divided into full-length sequences and non-full-length sequences. Based on the poly-A tail signal and the 5′ and 3′ cDNA primers in the ROIs, FLNC transcripts were identified [78]. The ICE (Isoform-clustering) algorithm in SMRT Analysis (v2.3.0) software was used to obtain a consistent sequence isoform, and the full-length consensus sequences were polished using Quiver. Full-length transcripts with a post correction accuracy above 99% were generated for further analysis. The redundant sequences were removed from the high-quality and corrected low-quality transcript sequences of each sample using CD-HIT (v4.6.8) software [79].

### Differential expression analyses

Analysis of transcript expression levels used a fragments per kilobase of transcript per million mapped reads (FPKM) method. Differential expression analysis was performed using the DESeq R package (Version 1.18.0). The *P* values were adjusted using the Benjamini & Hochberg method. A corrected *P*-value of 0.05 and log2 (fold change) of 1 were set as the thresholds for significant differential expression.

GO enrichment analysis of DETs or lncRNA target genes was performed by the GO seq R package [80], in which gene length bias was corrected. GO terms with corrected *P*-values less than 0.05 were considered significantly enriched for differentially expressed genes. KEGG is a database resource for understanding the high-level functions and utilities of biological systems, such as cells, organisms, and ecosystems [81], from molecular level information, especially large-scale molecular datasets generated by genome sequencing and other high-throughput experimental technologies (http://www.genome.jp/kegg/). KOBAS (v.3.0) software was used to test the statistical enrichment of DETs in KEGG pathways [82]. Plant transcription factors were predicted using iTAK (v1.7A) software [83].

### Quantitative real-time PCR analysis of DETs

The qRT-PCR assays were performed to validate the consistency of RNA-Seq analysis. QRT-PCR analysis was performed using a Light Cycler 480 machine with SYBR Green I Master Mix (TaKaRa, China). Three biological replicates were set for each treatment. The relative expression of each transcript was calculated after normalization to the reference gene. The relative quantification from three biological replications was normalized to the reference gene and was calculated by the 2^-ΔΔCt^ method. All primer sequences are shown in Additional file 15. Three technical replicates were performed based on the 2^-ΔΔCt^ algorithm using *EF1α* as a quantitative control.

### Statistical analysis

Statistical analysis was performed using ANOVA, and then a new multirange test was performed using Duncan’s range test in SPSS version 16.0. A significance level of *P* < 0.05 was applied to indicate significant values. Three independent biological replicates were used for RNA-seq and physiological and hormonal assays. Graphs were created using Origin 8.0 (Version 9.1; Origin Lab, Northampton, MA, USA).

## Supplementary Information

**Additional file 1: Fig. S1.** Characterization of *M. sieversii* transcripts. (A) The number of consensus reads in different lengths; (B) Novel gene statistical chart of annotation results of seven databases (NR, SwissProt, KEGG, KOG/COG, GO, NT, PFAM).

**Additional file 2: Fig. S2.** GO and KEGG enrichment analysis for 2234 DEGs. A: GO enrichment analysis for 2234 DEGs. B: KEGG enrichment analysis for 2234 DEGs.

**Additional file 3: Fig. S3.** CIRCOS visualization. The order of Circos from outside to inside are: **a.** Chromosomes of M.domestica. **b.** AS position. (Stacking histogram, turquoise: RI; green: A3; yellow: A5; purple: SE; red: MX; brown: AF; dark blue: AL). **c.** APA position mapped to chromosomes. **d.** Novel transcript density. **e.** Novel gene density. **f.** LncRNA density distribution. **g.** Fusion transcript distribution. Intrachromosome (purple); inter-chromosome (yellow).

**Additional file 4: Fig. S4.** Analysis of differentially expressed transcripts (DETs). (A) KEGG pathway enrichment on 1 h vs CK. (B) KEGG pathway enrichment on 6 h vs CK. (C) KEGG pathway enrichment on common DETs at different time points.

**Additional file 5: Fig. S5.** Validation of expression pattern of DEGs by qRT-PCR. Expression levels were analyzed using a T-test. Within each figure, asterisks above the bars indicate statistical significance (*p < 0.05; **p < 0.01). The normalized expression level (FPKM) of Illumina sequencing is indicated on the left y-axis and the relative expression of qRT-PCR is indicated on the right y-axis. Three biological replicates were set for each treatment. Experiments were repeated at least three times with similar results.

**Additional file 6: Table S1.** Details regarding AS.

**Additional file 7: Table S2.** Details regarding fusion gene.

**Additional file 8: Table S3.** Details regarding all DETs.

**Additional file 9: Table S4.** List of 2234 DEGs.

**Additional file 10: Table S5.** List of 4410 DETs.

**Additional file 11: Table S6.** GO enrichment analysis for 4410 DETs.

**Additional file 12: Table S7.** KEGG enrichment analysis for 4410 DETs.

**Additional file 13: Table S8.** Details regarding pathways.

**Additional file 14: Table S9.** Details regarding TFs.

**Additional file 15: Table S10.** Primers used for RT-qPCR verification.

## Data Availability

The sequence data have been deposited in the NCBI under BioProject accession number PRJNA706196 (https://dataview.ncbi.nlm.nih.gov/object/PRJNA706196?reviewer=dm9b28i3sjt7q3m8mngn3npnal).

## Abbreviations

CA: Cold acclimation
DEG: Differentially expressed gene
JA: Jasmonic acid
ABA: Abscisic acid
GO: Gene ontology
KEGG: Kyoto Encyclopedia of Genes and Genomes
FPKM: Fragments per kilobase of transcript per million mapped reads
FLNC: Full-length non-chimeric reads
Iso-Seq: Isoform Sequencing
CCS: Circular consensus sequence
REC: Relative electrolyte leakage values
Fv/Fm: Maximum photosynthetic efficiency values
GMAP: Genomic Mapping and Alignment Program
AS: Alternative splicing
DETs: Differentially expressed transcripts
