# Supplementary figures and images for: Transcriptome profiling of Malus sieversii under freezing stress after being cold-acclimated
Source: BMC Genomics. 2021 Sep 21;22:681. doi: 10.1186/s12864-021-07998-0 (PMC8456659; doi:10.1186/s12864-021-07998-0)

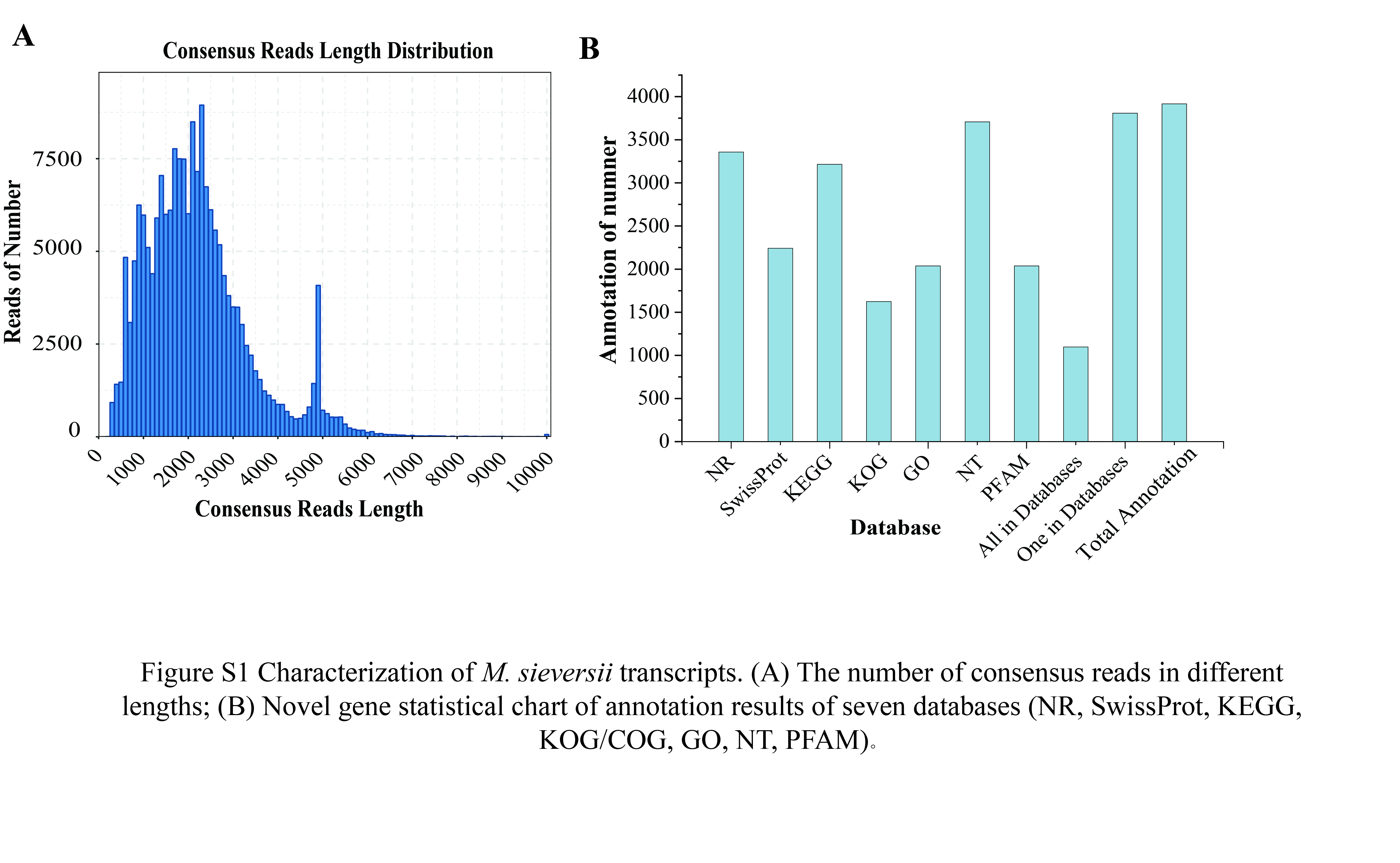

Supplement: Supplementary file 1 — Additional file 1: Fig. S1. Characterization of M. sieversii transcripts. (A) The number of consensus reads in different lengths; (B) Novel gene statistical chart of annotation results of seven databases (NR, SwissProt, KEGG, KOG/COG, GO, NT, PFAM). [file 12864_2021_7998_MOESM1_ESM.jpg]

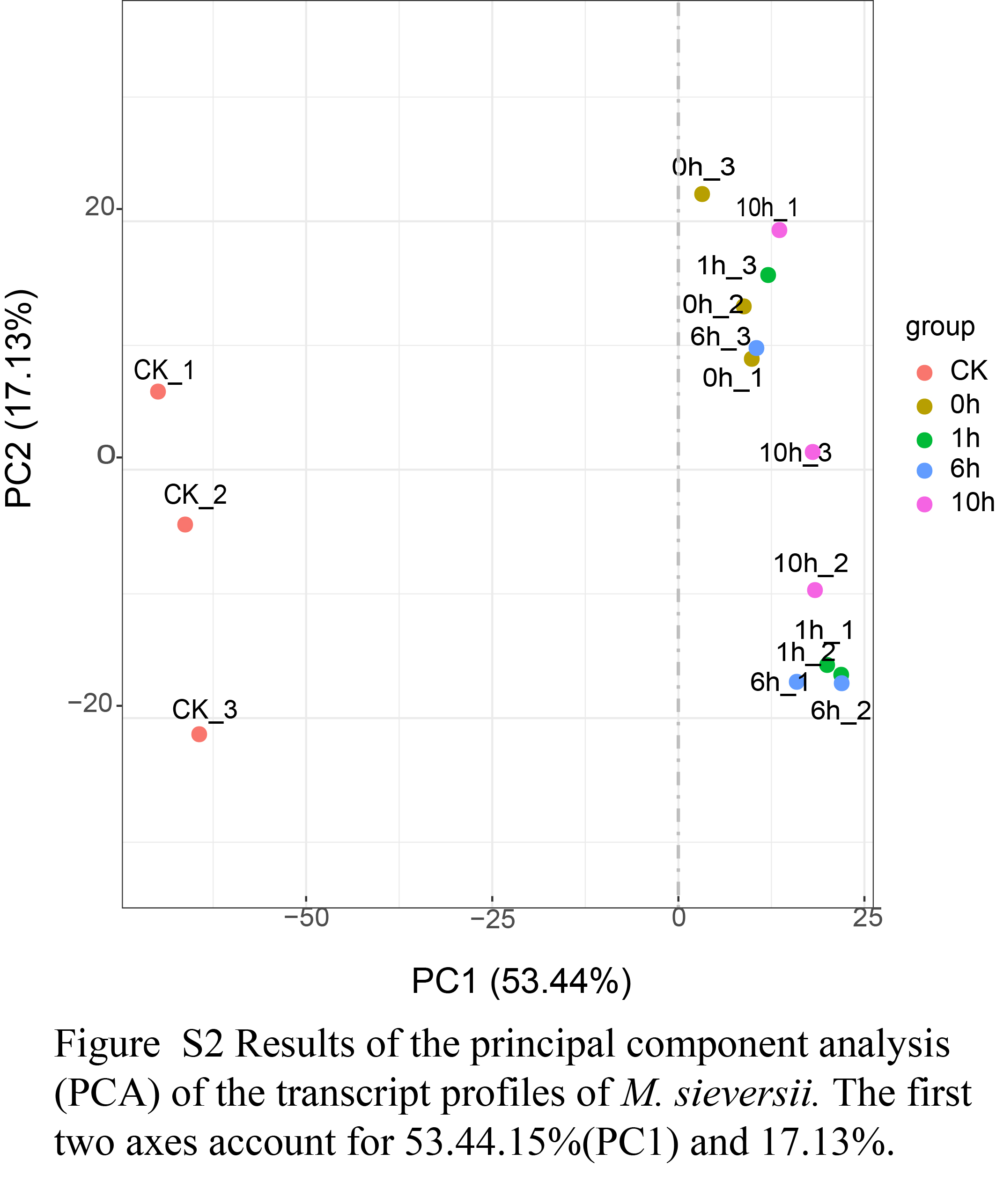

Supplement: Supplementary file 2 — Additional file 2: Fig. S2. GO and KEGG enrichment analysis for 2234 DEGs. A: GO enrichment analysis for 2234 DEGs. B: KEGG enrichment analysis for 2234 DEGs. [file 12864_2021_7998_MOESM2_ESM.jpg]

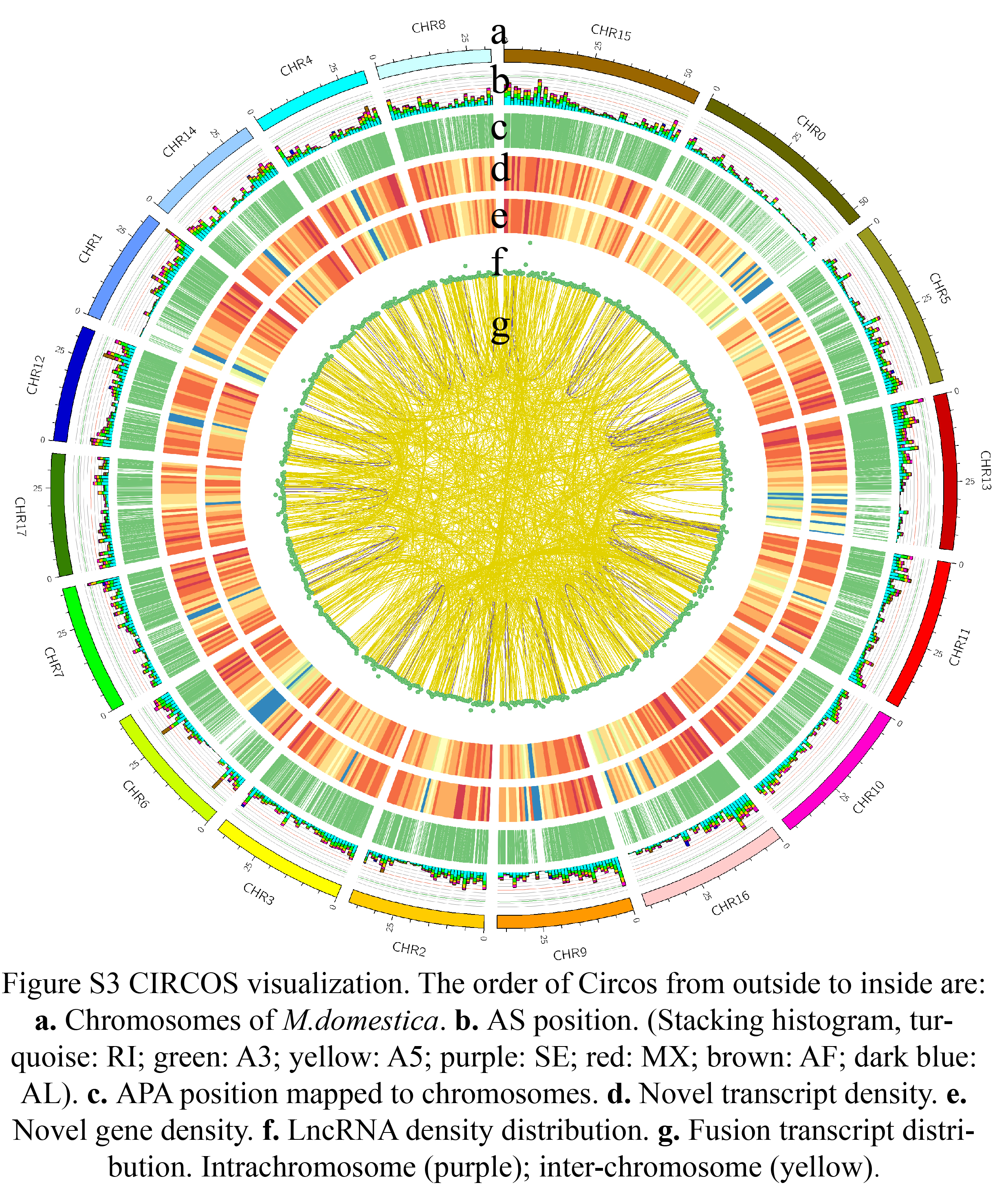

Supplement: Supplementary file 3 — Additional file 3: Fig. S3. CIRCOS visualization. The order of Circos from outside to inside are: a. Chromosomes of M.domestica. b. AS position. (Stacking histogram, turquoise: RI; green: A3; yellow: A5; purple: SE; red: MX; brown: AF; dark blue: AL). c. APA position mapped to chromosomes. d. Novel transcript density. e. Novel gene density. f. LncRNA density distribution. g. Fusion transcript distribution. Intrachromosome (purple); inter-chromosome (yellow). [file 12864_2021_7998_MOESM3_ESM.jpg]

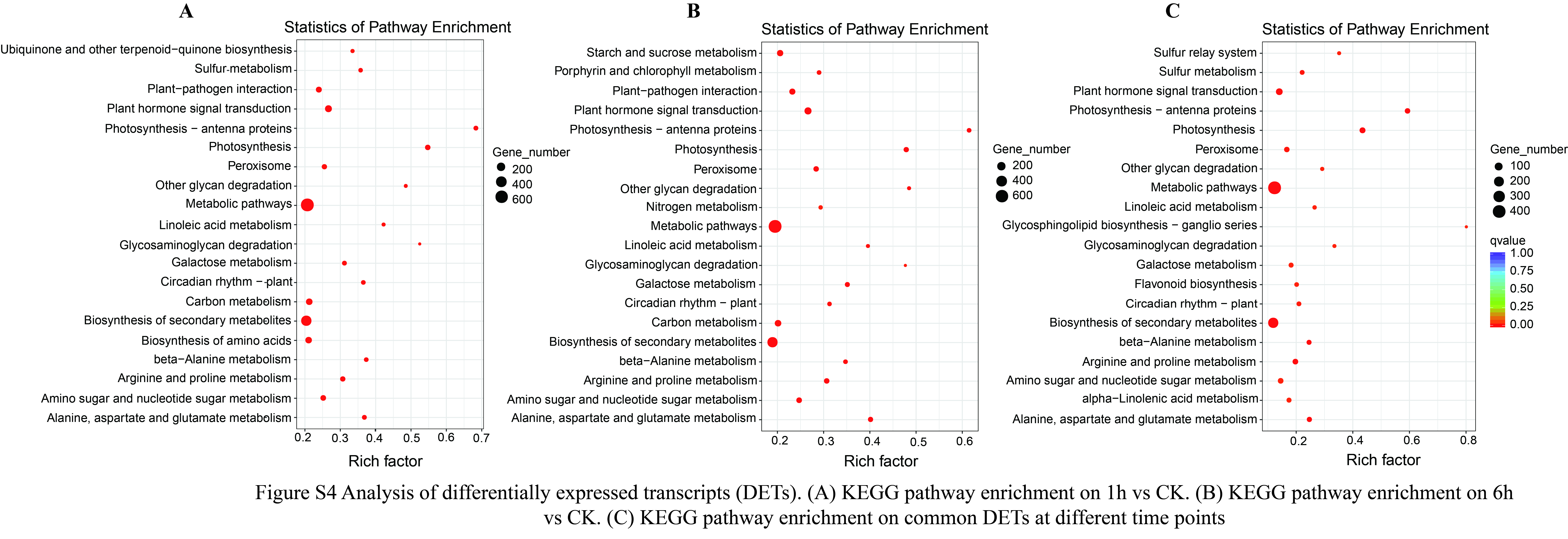

Supplement: Supplementary file 4 — Additional file 4: Fig. S4. Analysis of differentially expressed transcripts (DETs). (A) KEGG pathway enrichment on 1 h vs CK. (B) KEGG pathway enrichment on 6 h vs CK. (C) KEGG pathway enrichment on common DETs at different time points. [file 12864_2021_7998_MOESM4_ESM.jpg]

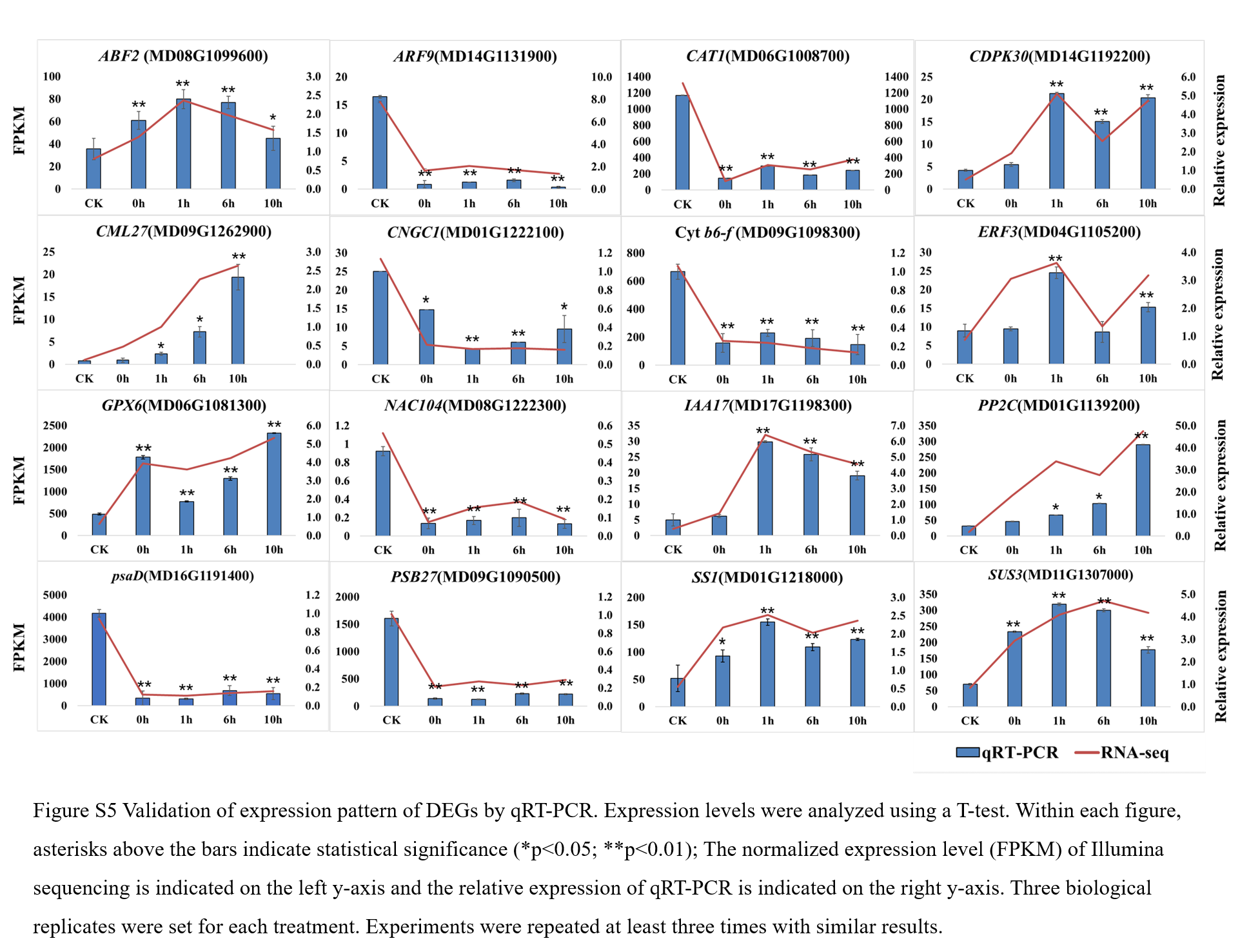

Supplement: Supplementary file 5 — Additional file 5: Fig. S5. Validation of expression pattern of DEGs by qRT-PCR. Expression levels were analyzed using a T-test. Within each figure, asterisks above the bars indicate statistical significance (*p < 0.05; **p < 0.01). The normalized expression level (FPKM) of Illumina sequencing is indicated on the left y-axis and the relative expression of qRT-PCR is indicated on the right y-axis. Three biological replicates were set for each treatment. Experiments were repeated at least three times with similar results. [file 12864_2021_7998_MOESM5_ESM.png]
