# Supplementary material for: Transcriptome profiling of Malus sieversii under freezing stress after being cold-acclimated
Source: BMC Genomics. 2021 Sep 21;22:681. doi: 10.1186/s12864-021-07998-0 (PMC8456659; doi:10.1186/s12864-021-07998-0)
Supplement: Supplementary file 15 — Additional file 15: Table S10. Primers used for RT-qPCR verification. [file 12864_2021_7998_MOESM15_ESM.docx]

Table S10 Primers used for RT-qPCR verification

| **gene ID** | **Annotation** | **Primers (5'to3')** |
| --- | --- | --- |
| MD01G1218000 | Starch synthase 1 | F: TCTGGTGGTTTAGAACTGGGAA  R: CACAAACAAGGAACCACGCA |
| MD11G1307000 | Sucrose synthase 3 | F: ATCGAGTGAGCAACGGTGAG  R: TGGAAGGCCGCAAGTCATAG |
| MD06G1008700 | CAT1 | F: TAACGTGAGGCCGAGCATTT  R: TGGCACCAAACAGGGATTACA |
| MD06G1081300 | GPX6 | F: GCATCTTGTAAGGGTTGCGTT  R: CGCATTGATATGACTGAATACTCGC |
| MD04G1105200 | ERF3 | F: CGGACTCTTTAGGGAGGCAA  R: CGAAGGCAAAGATACCGCAG |
| MD08G1222300 | NAC104 | F: CAACCCAAAACAGAATACAGTGG  R: AAGAACACTTCGTCCAAGCAT |
| MD09G1098300 | cytochrome b6-f complex | F: TCAGAACCGGCGAAGATCCA  R: GGTCCTCCTTGCACACACAAA |
| MD16G1191400 | photosystem I reaction center subunit II | F: CATTACCCCATAACCCCCGA  R: GTGAAGAGGCTGGCTTGTGT |
| MD09G1090500 | PSB27 | F: ATGGAGAGATGAGAGAGGAGCA  R: AGAGGGTTTTCATCCCCACC |
| MD01G1222100 | CNGC1 | F: CCTTCCTTCGTTTCCGACCA  R: GCGTCTCTGCTTTGACGTTT |
| MD09G1262900 | CML27 | F: AAGTGGGTGGCCGTAACAAA  R: CAATTCCGCGTTGAGGTCAG |
| MD14G1192200 | CDPK30 | F: GGCAGGCGATTTGTTTCTTCA  R: GAGAAAAGAGGCGTGCACAAAT |
| MD17G1198300 | IAA17 | F: GGAAACATGCAAGCGACTCC  R: TCCCCAAACAACACGAGAGAG |
| MD14G1131900 | ARF9 | F: GTCCTGTGTTCGGTAGTGGG  R: TCCCGTCACTATCCACCAAAC |
| MD01G1139200 | PP2C | F: AGCGTTGTTGTCGTGGATCT  R: ATTTCCTGCTTACACGGGGG |
| MD08G1099600 | ABF2 | F: AACCCAATTCTGTCTACCCCTT  R: TCAGCAACACACAGCAAGAATC |
| MDP0000470429 | EF1α | F: AGGATGCTACAGCCGATGAG  R: GCCGAAGAACTGACGAGAATC |
